# Haplotypes, Genotypes, and DNA Methylation Levels of Neuromedin U Gene Are Associated with Cardio-Metabolic Parameters: Results from the Moli-sani Study

**DOI:** 10.3390/biomedicines13081906

**Published:** 2025-08-05

**Authors:** Fabrizia Noro, Annalisa Marotta, Simona Costanzo, Benedetta Izzi, Alessandro Gialluisi, Amalia De Curtis, Antonietta Pepe, Sarah Grossi, Augusto Di Castelnuovo, Chiara Cerletti, Maria Benedetta Donati, Giovanni de Gaetano, Francesco Gianfagna, Licia Iacoviello

**Affiliations:** 1 Research Unit of Epidemiology and Prevention, IRCCS NEUROMED, 86077 Pozzilli, IS, Italy; 2 EPIMED Research Center, Department of Medicine and Surgery, University of Insubria, 21100 Varese, VA, Italy; 3 Department of Medicine and Surgery, LUM University, 70010 Casamassima, BA, Italy; 4 Health and Socio-Health Direction, ASST Sette Laghi, 21100 Varese, VA, Italy

**Keywords:** neuropeptides, gut–brain axis, coronary diseases, cerebrovascular disease

## Abstract

**Background/Objectives:** Neuromedin U (NMU) is a highly conserved gene encoding a neuropeptide involved in the regulation of feeding behavior and energy homeostasis. We aimed to analyze the association between *NMU* genetic and epigenetic variations and cardio-metabolic parameters in an Italian population to identify the role of these variants in cardio-metabolic risk. **Methods:** A total of 4028 subjects were randomly selected from the Moli-sani study cohort. *NMU* haplotypes were estimated using seven SNPs located in the gene body and in the promoter region; DNA methylation levels in the promoter region, previously associated with lipid-related variables in the same population, were also used. **Results**: Among the haplotypes inferred, the haplotype carrying the highest number of minor variants (frequency 16.6%), when compared with the most frequent haplotype, was positively associated with insulin levels, HOMA-IR, and diastolic blood pressure, and negatively with HDL-cholesterol. The multivariable analysis that considered methylation levels along with their interactions with SNPs showed that increased methylation levels in two close CpG sites were associated with higher levels of lipid-related variables. **Conclusions**: This study supports a role for NMU as a regulator of human metabolism. This finding suggests that NMU could be a potential target for preventive interventions against coronary and cerebrovascular diseases, and that *NMU* genetic and epigenetic variability may serve as a biomarker for cardio-metabolic risk.

## 1. Introduction

Metabolic disorders, such as insulin resistance, abdominal obesity, dyslipidemia, and hypertension, frequently occur together and are associated with an increased cardiovascular disease (CVD) risk [1]. An underlying pro-inflammatory condition frequently coexisting with these phenotypes, called metabolic inflammation, is considered the main reason for the increased CVD risk [2]. A chronic low-grade pro-inflammatory state often coexisting with these conditions, referred to as metabolic inflammation, is considered a major contributor to the elevated CVD risk [2]. Metabolic and inflammatory stress promote the onset and progression of CVD by activating complex molecular pathways that lead to endothelial dysfunction, plaque instability, and tissue damage [3]. However, the precise mechanisms linking metabolic disorders and inflammation are not yet fully understood [4,5].

Neuromedin U (NMU) is a neuropeptide expressed in several peripheral tissues that could be considered a potential bridge between metabolic disorders and inflammation and a good candidate as a biomarker in CVD risk assessment [6,7]. In fact, studies on murine models showed pleiotropic roles for this peptide in metabolic functions and inflammation, as well as in the regulation of blood pressure, hormone release, and tumorigenesis [8,9,10]. In addition to its peripheral actions, NMU is also highly expressed in the central nervous system, particularly in the hypothalamic paraventricular and dorsomedial nuclei, where it plays a key role in regulating feeding behavior, energy expenditure, and circadian rhythms [11,12]. The central administration of NMU has been shown to exert anorexigenic effects, to suppress food intake, and to activate the hypothalamic–pituitary–adrenal (HPA) axis [13,14]. Studies in mice also indicate that NMU plays a role in stress response, anxiety-like behaviors, and locomotor activity [15].

In humans, NMU has been found to be involved in inflammatory and immune processes [16]. Our group also reported a role of this neuropeptide in potentiating platelet activation induced by several agonists like adenosine diphosphate, epinephrine, and serotonin [17]. Beyond experimental models, population studies on humans showed associations of *NMU* genetic variants with overweight and obesity in children and adults [18,19,20] as well as with food preferences [21] and bone density [22] in children. We have recently found *NMU* promoter methylation to be associated with metabolic and inflammatory indices in a cohort of adults [23].

In this study, we aimed at investigating the association between *NMU* genetic and epigenetic variability and several cardio-metabolic risk factors in an Italian general adult population. We firstly used a haplotype analysis approach, which allowed us to capture most of the existing combinations of genetic variants found in a population (haplotypes) confined to specific regions. We repeated the analyses, adding DNA methylation levels at single CpG sites.

## 2. Materials and Methods

### 2.1. Study Population

The study population was composed of a random subsample of 4028 participants from the Moli-sani study, a prospective cohort study established in 2005–2010 with an enrolment of 24,325 men and women (aged ≥ 35 years) randomly recruited from the general population of Molise, a Southern Mediterranean Italian region, with the purpose of investigating risk factors in the onset of cancer and cardiovascular and cerebrovascular diseases. Exclusion criteria were pregnancy at the time of recruitment, mental impairments, current polytraumas or coma, or refusal to sign the informed consent [24,25,26].

The Moli-sani study complies with the Declaration of Helsinki and was approved by the Ethical Committee of the Catholic University of Rome, Italy. All participants provided written informed consent.

The subsample size was selected to study the associations between continuous metabolic variables and haplotypes at a frequency higher than 10% to obtain adequate statistical power based on the expected results according to the literature. Subjects with unreliable questionnaires or with missing values for core variables were excluded.

The analyzed sub-cohort and the whole Moli-sani population showed similar values in all variables considered for the present study (Supplementary Table S1).

### 2.2. Data Collection

Detailed, structured questionnaires on medical history and lifestyles were administered to all subjects. A full description of the collected data is reported elsewhere [23].

Blood pressure (mm Hg) was measured by an automatic device (OMRON HEM-705CP, OMRON Healthcare, Shimogyo-ku, Kyoto, Japan) three times on the non-dominant arm, with the patient lying down for about 5 min. The second and the third measurements were averaged to compute the final measure. Body mass index (BMI) was calculated as weight (kg)/height (m)^2^. Waist and hip circumferences were measured according to the National Institutes of Health, Heart, Lung, and Blood Guidelines [27] to calculate the waist-to-hip ratio. Metabolic syndrome was defined according to Adult Treatment Panel III criteria [28].

### 2.3. Biochemical Analyses

Blood samples were obtained between 07:00 and 09:00 from participants who had fasted overnight and had refrained from smoking for at least 6 h.

Hemochromocytometric analyses were performed on freshly collected blood by a cell counter (Coulter HMX, Beckman Coulter, IL, Milan, Italy) within 3 h of venipuncture.

Serum lipids and glucose were assayed by enzymatic reaction methods using an automatic analyzer (ILab 350, Instrumentation Laboratory, Milan, Italy). The concentration of low-density lipoprotein (LDL) cholesterol was calculated using the Friedewald formula. These analyses were performed in the centralized Moli-sani laboratory. Insulin and apolipoprotein A-I and B measurements were performed using automated immunoassay on samples frozen in nitrogen vapor at Neuromed Biobanking Center and subsequently transferred to the BiomarCaRE project centralized laboratory [29]. The HOMA-IR (homeostatic model assessment of insulin resistance) was calculated as insulin (mU/L)*glucose (mg/dL)/405 [30].

### 2.4. NMU Genetic Variant Analysis

*NMU* genotyping was performed on white blood cell DNA. Buffy coats of peripheral blood cells were freshly isolated from whole blood samples collected in sodium citrate and EDTA by centrifugation at 3000 rpm for 20 min at room temperature, frozen in nitrogen vapor, and stored at Neuromed Biobanking Centre until utilized (http://www.neuromed.it/biobanking-centre/, accessed on 12 April 2025) [31]. DNA was subsequently extracted using a silica matrix-based method, as previously described [32], its concentration verified with NanoDrop spectrophotometry, and samples stored at −20 °C until analyzed.

**Figure 1:**
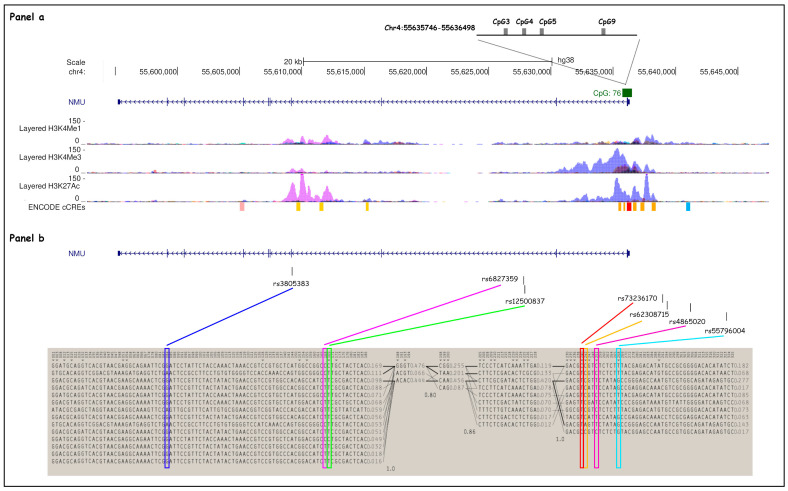
Selected NMU SNP and haplotype block structures. (**Panel a**) *NMU* (chr4: 55,595,231–55,636,298; GRCh38/hg38 assembly) spans chromosome region 4q12 and is composed of nine exons (depicted as blue lines throughout the gene). H3K4Me1, H3K4Me3, and H3K27Ac profiles are displayed as colored overlaid histograms (Bernstein Laboratory at the Broad Institute and the University of California, Santa Cruz, and part of the ENCODE database). Four CpG sites from the CpG island 76 (chr4: 55,635,746–55,636,498, GRCh38/hg38 assembly) found to be significantly associated with methylation patterns in Marotta et al. 23. are shown as gray boxes. (**Panel b**) Seven SNPs (depicted as black lines on the top) were selected for the haplotype study: rs73236170, rs62308715, rs4865020, and rs55796004, located upstream of the *NMU* TSS, and rs3805383, rs6827359, and rs12500837 within the gene body. Two main haplotype blocks (chr4: 55,595,229–55,653,833; GRCh38/hg38 assembly) have been identified in *NMU*, in the gene body (block on the left) and in the promoter region (block on the right), respectively.

*NMU* spans chromosome region 4q12 (chr4: 55,595,231–55,636,298; GRCh38/hg38 Assembly), and it is composed of 9 exons. Two main *NMU* haplotype blocks were selected from Hapmap Project (CEU, TSI populations) and 1000 Genomes Project data using the Tagger Pairwise method on Haploview software (version 4.2; Broad Institute, Cambridge, MA, USA) [33] (Figure 1); one in the gene body (GTT, GCC, GTT, GCT, ATT)—tagged by rs3805383, rs6827359, rs12500837—and the other in the promoter region (TCTG, TCCT, CCCT, TATG, and TCTT), tagged by rs73236170, rs62308715, rs4865020 and rs55796004.

The SNP selection was based on the following criteria.

First, a set of SNP lists defining haplotypes with frequency higher than 10% was selected.

Second, we evaluated SNP type and position in the gene using the Genome Variation Server database as well as the correlation between SNPs in the same haplotype block (linkage disequilibrium, pairwise r2 threshold of 0.8).

Third, we considered SNPs that were previously associated with metabolic phenotypes in GWAS studies retrieved from literature or large genetic databases (GWAS Central, OMICS db in PubMed).

Moreover, we selected variants that could have a regulatory role in *NMU* gene expression. In particular, rs4865020 co-localizes with a CTCF binding region according to the ENCODE Candidate Cis-Regulatory Elements database and with moderate deposition of H3K4Me1 histone mark (Supplementary Figure S1).

Concerning the SNPs selected within the gene body, rs3805383 tags a region where there is a high presence of H3K27Ac and moderate presence of H3K4Me1 histone modifications, usually associated with transcriptional activation. Located at around 400 bp from another distal enhancer element, this SNP also tags a highly conserved region across species (Supplementary Figure S2).

The SNP set was enriched through imputation using the CEU and TSI populations from the Hapmap Project and 1000 Genomes Project as a reference set.

*NMU* variants were genotyped using a 7500 Fast Real-Time System (Applied Biosystems, Thermo Fisher Scientific, Waltham, MA, USA) with standard Taqman reagents and protocols. Positive and negative controls (homozygous allele 1, homozygous allele 2, heterozygous, no template control) were selected from the cohort.

Genotyping data were obtained by using the SDS v1.4 software (Applied Biosystems, Thermo Fisher Scientific, Waltham, MA, USA). Primers and probes used in the assay for each SNP are listed in Supplementary Table S2. The quality of the genotyping was assured by Hardy–Weinberg equilibrium (HWE, Chi-squared *p* > 0.05), concordance with population frequencies (Hapmap CEU and TSI, 1000 Genomes Project), concordance between duplicate samples (a locus for each DNA sample was repeated twice), and frequencies of rare haplotypes, as estimated from the software.

### 2.5. NMU DNA Methylation Analysis

Detailed methods were previously reported [21]. Briefly, *NMU* methylation analysis was performed on white blood cell DNA using the Pyrosequencer Q48 Autoprep (QIAGEN) platform using peripheral blood cells from a subsample of 1211 subjects included in this study. Amplicons covering 10 CpG sites from CpG island 76 (chr4: 55,635,746–55,636,498) in the promoter region were considered. All PCR amplifications were performed in duplicate. The two assays were tested using fully methylated and unmethylated controls (EpiTect PCR Control DNA Set, QIAGEN, Hilden, Germany). For the CpG-specific analysis, data were discarded when the duplicate measurements had a standard deviation (SD) ≥ 5%.

### 2.6. Statistical Analysis

All analyses were performed using R software (v3.2.1; https://www.R-project.org/, accessed on 12 April 2025) and SAS software (Version 9.4 for Windows©2009. SAS Institute Inc., Cary, NC, USA). Mean and SD were computed for continuous variables (z-scores or log-transformed variables were used where appropriate) and frequencies for categorical variables (Table 1).

Regression analyses were performed to evaluate associations between haplotypes and cardio-metabolic indices, using age and sex as covariates. The R Haplo.stats package (https://CRAN.R-project.org/package=haplo.stats, accessed on 12 April 2025) was used to estimate the haplotype frequencies based on the genotyped SNPs and to verify the associations between haplotypes and phenotypes (haplo.glm function, the most prevalent haplotype, was used for reference). Haplotypes with frequencies lower than 1% were excluded from the analysis.

The same analysis was then repeated using all the available genotypes in models including (i) single SNPs or (ii) all the available SNPs, along with age and sex. For the analysis including all the SNPs, a stepwise model with backward elimination was used to finally retain only the statistically significant associated genotypes.

We imputed the missing genotypes (n = 24 to 226 for the seven SNPs, Table 2 and Table 3) using the estimation function (haplo.em), which deals with sparse missing genotypes, imputing them based on the linkage found in the case-complete subsample. Moreover, using SAS/STAT software software (version 9.4, SAS Institute Inc., Cary, NC, USA), we checked the Hapmap Project (CEU, TSI populations) and 1000 Genomes Project databases to identify and impute any other unmeasured SNPs tagging the haplotypes, or combinations of them, which were retrieved using combinations of measured tag SNPs.

The best genetic model was checked for each haplotype/genotype–phenotype association using dominant, codominant, and recessive models for the analysis. The codominant/additive model was the best genetic model (i.e., the one with the smallest AIC) for the majority of the genotype–phenotype associations and thus was used for all subsequent analyses. A Benjamini–Hochberg false discovery rate (FDR) was used to adjust the results for multiple comparisons, accounting for a total of 13 phenotypes and a single haplotype block. FDR-adjusted *p*-values (pFDR) < 0.05 were considered statistically significant.

Since lipid-related variables were found to be associated with DNA methylation patterns of the NMU promoter region in the same study population [21], the analyses for these phenotypes were repeated, including both genotypes and the CpG methylation levels resulting from association (CpG3, CpG4, CpG5, CpG9).

Once we identified the genetic variants remaining statistically significant associated, the interactions between pairs of the associated SNPs and CpG sites were tested in models including the interaction and its constitutive terms. The significant interactions were then added to the final model.

## 3. Results

Following the genetic analysis and the related quality control of concordance between genotypes of duplicate samples, we excluded the 75 subjects with discordant results, leading to a final study population of 3953 subjects. Baseline characteristics are described in Table 1. The mean age was 55.7 years (±12.9 SD), and 48.7% were male. The prevalence of diabetes, hypertension, and dyslipidaemia was 9.8%, 58.1%, and 33.7%, respectively. Participants with metabolic syndrome accounted for 28.1% of the studied population.

All genotyped SNPs were in Hardy–Weinberg equilibrium (HWE), and the minor allele frequencies (MAFs) were similar to values reported in the HapMap Project database for the Tuscan (TSI) and Caucasian (CEU) populations (Supplementary Table S3).

Using data from the seven selected tag SNPs and considering the whole region comprising the two selected haplotype blocks, eight haplotypes (H1–H8) were inferred (Table 2).

Four additional SNPs were imputed, based on linkage with the retrieved haplotypes, allowing for coverage of the tract variability for 11 SNPs. The most frequent haplotype (H1, 25.2%) carried only one minor allele out of 11 loci, while the others included between one and five minor alleles. Genotype frequencies are reported in Table 3. The list of all SNPs in linkage disequilibrium (LD) with the 11 genotypes is reported in Supplementary Table S4.

### 3.1. Association Between Haplotypes and Metabolic Indices

Table 4 shows the associations between cardio-metabolic variables and haplotypes with a frequency greater than 10%, each carrying a different number of minor alleles, using the most frequent haplotype for reference (H1, including only one minor allele).

The analysis considering the contrast with the haplotype carrying the highest number of minor alleles (H8, five minor alleles, frequency 16.6%) showed a positive association between haplotype copy number and glucose metabolism-related variables, in particular with insulin levels (standardized variable, β ± SE = 0.11 ± 0.04, *p* = 0.002) and the derived variable HOMA-IR (0.09 ± 0.03, *p* = 0.013).

A positive association was also found with diastolic (0.09 ± 0.03, *p* = 0.008) but not systolic blood pressure. Regarding lipid metabolism, a negative association with HDL-cholesterol (−0.09 ± 0.03, *p* = 0.006) was found. These associations survived FDR correction.

The other haplotypes did not show FDR-significant associations, although a nominally significant association was found for H8 with hypercholesterolemia (OR = 0.82, 95% CI = 0.71–0.96). Supplementary Table S5 reports the results for haplotypes with a frequency lower than 10%.

### 3.2. Association Between SNPs and Metabolic Indices

Table 5 reports the genotypes associated with the cardio-metabolic variables, including those resulting in statistically significant differences in the previous haplotype analysis and in the previous DNA methylation analyses (lipid-related variables) performed in the same study population [21].

Only the SNPs that survived backward elimination of non-significant results have been reported. As for insulin and the HOMA index, beyond rs28451532 tagging the H2 haplotype (standardized variable, β ± SE = 0.09 ± 0.03, *p* = 0.003, and 0.08 ± 0.03, *p* = 0.006, respectively), rs12501006 (H5—haplotype frequency 3.2%, and rare haplotypes) was also associated (0.12 ± 0.06, *p* = 0.048, and 0.18 ± 0.06, *p* = 0.002). As for lipids, five SNPs located in the coding region of *NMU* were retained in the final model.

Very similar results were observed for total, LDL-, and HDL-cholesterol levels, which were associated positively with rs11945489 and rs3805383 and negatively with rs140220080 and rs6827359. Non-HDL variables and apolipoprotein B were also positively associated with rs73236170. No associations were found with diastolic blood pressure. A full list of results for all studied variables, for models including a single variant in each model or the whole set of genotypes, is reported in Supplementary Table S6a,b.

### 3.3. Association Between SNPs, CpG Methylation Levels, and Metabolic Indices

Table 6 reports the results of the models investigating the association between both genetic variants and CpG sites with lipid-related variables, which were found to be significantly associated with NMU methylation in our previous work [21].

First, we used multivariable models and, following a backward elimination of non-significant variables, we found that both SNPs and CpGs remained statistically significant in all analyses (R-squared from 0.034 to 0.045). In the analysis including the interaction terms between SNP–CpG pairs, those between the SNPs rs73236170 or rs3805383 and CpG sites 4 or 5 were also retained in the final models (R-squared improved by 0.01 for LDL-cholesterol and apolipoprotein B). Supplementary Table S7 reports the associations between genetic and epigenetic variants, showing no significant associations.

## 4. Discussion

We report the relation between *NMU* genetic variability and a number of cardio-metabolic parameters in a general population of Italian adults using a haplotype-based approach. The main result is the identification of an *NMU* haplotype, ACTCCCT (haplotype frequency = 16.6%), significantly associated with increased insulin levels and the derived variable HOMA-IR, as well as with increased diastolic blood pressure and decreased HDL-cholesterol levels. Following our previous results on the associations between DNA methylation patterns at CpG sites located in the *NMU* promoter and lipid-related variables, in this study, we validated and replicated our findings using a combination of SNPs and specific CpG sites that were both found to explain lipid-related variability.

Our results support the role of NMU in the regulation of insulin production/release, previously observed in experimental studies [34], and suggest a potential influence of NMU in the development of insulin sensitivity and related metabolic disorders. We found associations between genetic variants linked with the H8 haplotype and insulin resistance, measured through insulin levels and the HOMA index. This finding was previously observed in studies on transgenic mouse models [35]. NMU plays a crucial role in glucose homeostasis. Although NMU treatment of isolated human islets suppresses insulin production and secretion, acting like a decretin hormone [36], it also appears to improve glucose tolerance [37]. In fact, peripheral treatment with NMU results in elevated GLP-1 levels in mice and improved glucose tolerance via NMUR1 signaling [38]. All these effects could also be due to an indirect effect on glucose homeostasis, mediated by vagus-dependent reduction of gastric emptying following meals, and to the reduced peaks of nutrient absorption [39]. It is worth noting that NMU also exerts multiple central effects, which may influence metabolic phenotypes indirectly. NMU has been shown to suppress appetite, regulate circadian behavior, and modulate the stress response via central nervous system pathways. These neurobehavioral effects could interact with peripheral mechanisms [7,20]. Future studies combining laboratory and behavioral assessments with genetic and epigenetic profiling may help clarify how central and peripheral NMU functions converge in the pathogenesis of cardio-metabolic diseases.

Beyond the association with glucose-related parameters, we also found associations between the same genetic variants and other metabolic parameters. Insulin resistance is the main underlying condition of metabolic syndrome, since insulin has beneficial effects on blood pressure, metabolism of lipids, and cholesterol-binding lipoproteins [40,41]. This suggests that the effect on insulin may be the driving phenotype in the associations reported in this study.

Our haplotype analysis showed that the most significantly associated variants are mainly located in the gene body. This suggests a possible link with changes in mRNA structure and its processing. However, many of these variants are in linkage with others distributed across the gene region, including the upstream promoter of DNA methylation. These variants may act as regulatory elements [42,43]. Worth noting is rs3805383, which is linked to the H8 haplotype and found to be significantly associated with lipid-related variables in genotype association analyses. This variant co-localizes with an active enhancer since it is located in a region with high deposition of both H3K27Ac and H3K4Me1 histone marks [44] (Supplementary Figure S2). As a consequence, the presence of gene variants in these regions is likely to influence the enhancer activity, potentially leading to an altered gene expression [44].

Methods based on haplotypes are more powerful in disease gene mapping than those based on single variation markers [45]. Moreover, since *NMU* haplotype blocks are short and highly conserved across different species, a high impact on peptide function and/or transcription regulation was expected for human genetic variations of this tract. However, we took advantage of the tag SNPs measured and imputed; thus, we also performed genotype–phenotype associations. We identified variants in the promoter region associated with cholesterol (both HDL- and non-HDL cholesterol content in the same direction) and apolipoprotein B levels. In our previous study focusing on *NMU* methylation measured in a Moli-sani sub-cohort [23], which included some of the subjects involved in this analysis, we also found an association between *NMU* DNA methylation and lipid-related variables. Adding this methylation contribution to the genetic analyses, we interestingly found that both CpG methylation levels and SNPs, both located in the promoter region, as well as their interactions, were significantly associated with lipid-related parameters. Moreover, in our previous study, we also found an association between those CpG sites and inflammatory variables [23]. This finding suggests that NMU could be one of the factors linking inflammation and metabolic disorders in subjects with cardio-metabolic diseases. This result strengthened the suggestion to further study *NMU* and other genes belonging to its pathway as markers or as potential targets for interventions on lipid-related, inflammatory, and cardio-metabolic diseases.

The main strengths of this study are the availability of a large, homogenous population, which represents a good setting for genetic studies, and the wealth of standardized cardio-metabolic measures. We also acknowledge some limitations. Although a confounding effect should be excluded due to the genetic study design, we cannot rule out a modification of the genetic effect due to some factors not taken into account here, like diet or physical activity. A further limitation is the use of a single discovery cohort, without any replication. Caution is necessary in extending these results to larger population contexts, since data were collected in a single Italian region. However, the main characteristics of our population sample are comparable to those of the Italian Cardiovascular Epidemiology Observatory [46]. For this reason, our sample could be considered representative of at least the Italian population. In addition, a further limitation of our study is the absence of *NMU* gene variant-dependent expression analyses. Future studies leveraging CRISPR-based functional assays will be necessary to determine whether *NMU* variants directly modulate *NMU* transcription or influence it through alternative regulatory mechanisms.

## 5. Conclusions

These results suggest the presence of a functional genetic and epigenetic variant(s) of the neuromedin U gene influencing phenotypic variation in metabolic indices, encouraging further studies on *NMU* variants as biomarkers for risk assessment of cardio-metabolic disorders. Our study also opens up future investigation to characterize the functional role of the identified *NMU* variants. By integrating functional genetic and epigenetic approaches, it will be possible to better unravel the contribution of the neuropeptide NMU in the pathophysiology of cardio-metabolic disturbances including CVD.

## Figures and Tables

**Table 1:** Population characteristics (Moli-sani N 3953).

Continuous Variables	Mean	SD
Age (years)	55.7	12.1
Mediterranean diet score	4.3	1.6
Alcohol intake (g/day)	17.3	23.9
Energy intake (kcal/day)	2109	683
BMI (kg/m^2^)	28.11	4.63
Waist-to-hip ratio	0.93	0.08
Blood glucose (mg/dL)	102.26	25.10
Insulin (pmol/L)	57.89	41.76
HOMA-IR	2.17	1.88
Systolic blood pressure (mm Hg)	141.60	20.50
Diastolic blood pressure (mm Hg)	82.24	9.33
Total cholesterol (mg/dL)	216.32	41.24
LDL-cholesterol (mg/dL)	132.21	35.55
Apolipoprotein B (g/L)	0.97	0.23
HDL-cholesterol (mg/dL)	58.22	14.56
Apolipoprotein A-I (g/L)	1.54	0.31
Triglycerides (mg/dL)	131.97	84.85
Categorical variables	n	%
Sex (male)	1934	48.9
Ever smoked	1983	50.2
Overweight or obesity	2893	73.2
Diabetes mellitus	384	9.8
Hypertension	2280	58.0
Hypercholesterolemia	1304	33.5
Metabolic syndrome	1102	28.0
Previous CVD	207	5.3

Abbreviation: BMI: body mass index; CVD: cardiovascular disease; HDL: high-density lipoprotein HOMA-IR: homeostatic model assessment of insulin resistance; LDL: low-density lipoprotein; SD: standard deviation. Results are presented as means (standard deviation, SD) for continuous variables and as frequencies and percentages for categorical variables.

**Table 2:** Alleles and frequencies of the inferred haplotypes.

Minor Alleles, N		Haplotype *			n/n		n/H		H/H	
			N	Freq.	n	%	n	%	n	%
1	H1	[GAC]GTT[C]TCTG	3953	25.2%	2215	56.0	1486	37.6	252	6.4
1	H2	[GAC]GTT[C]TCCT	3953	6.0%	3499	88.5	435	11.0	19	0.5
2	H3	[GAC]GTT[C]TATG	3953	14.1%	2921	73.9	951	24.1	81	2.0
3	H4	[GAT]GCT[C]TCCT	3953	5.3%	3546	89.7	393	9.9	14	0.4
3	H5	[AAC]ATT[G]TCTT	3953	3.2%	3702	93.6	248	6.3	3	0.1
4	H6	[GAT]GCC[C]TCCT	3953	15.4%	2830	71.6	1029	26.0	94	2.4
4	H7	[GAT]GCC[C]TCTG	3953	10.0%	3199	80.9	715	18.1	39	1.0
5	H8	[GGC]ACT[C]CCCT	3953	16.6%	2768	70.0	1058	26.8	127	3.2
	Rare	Low-frequency haplotypes °	3953	4.2%	3635	91.9	303	7.7	15	0.4

* Alleles for the following ([imputed], measured) SNPs: [rs140220080, rs28451532, rs11945489], rs3805383, rs6827359, rs12500837, [rs12501006], rs73236170, rs62308715, rs4865020, rs55796004; underlined: minor alleles. ° Pool of haplotypes with frequency lower than 1%.

**Table 3:** Characteristics and frequencies of the available *NMU* genotypes (measured plus imputed).

			Homozygotes, Major		Heterozygotes		Homozygotes, Minor	
Genotype	N	Imputed	Freq.	%	Freq.	%	Freq.	%
[rs140220080]	3953	3953	3655	92.5	289	7.3	9	0.2
[rs28435401]	3953	3953	2733	69.1	1082	27.4	138	3.5
[rs11945489]	3953	3953	1829	46.3	1698	42.9	426	10.8
rs3805383	3953	226	2476	62.6	1281	32.4	196	5.0
rs6827359	3953	77	1032	26.1	1930	48.8	991	25.1
rs12500837	3953	223	2136	54.0	1537	38.9	280	7.1
[rs12501006]	3953	3953	3672	92.9	276	7.0	5	0.1
rs73236170	3953	32	2630	66.5	1169	29.6	154	3.9
rs62308715	3953	24	2910	73.6	960	24.3	83	2.1
rs4865020	3953	31	1180	29.9	1931	48.8	842	21.3
rs55796004	3953	34	1069	27.1	1938	49.0	946	23.9

Square brackets: completely imputed genotypes.

**Table 4:** Associations between *NMU* haplotypes from both internal and promoter regions and cardio-metabolic indices.

		H8 [GGC]ACT[C]CCCT ^§^ 5 Minor Alleles, 16.6%			HA6 [GAT]GCC[C]TCCT 4 Minor Alleles, 15.4%			H7 [GAT]GCC[C]TCTG 4 Minor Alleles, 10.0%			H3 [GAC]GTT[C]TATG 2 Minor Alleles, 14.1%		
	N	β *	SE	*p* °	β	SE	*p*	β	SE	*p*	β	SE	*p*
BMI	3949	0.081	0.035	0.021	0.002	0.037	0.96	0.076	0.044	0.083	0.046	0.037	0.22
Waist-to-hip ratio	3948	0.017	0.032	0.60	−0.090	0.034	0.008	−0.021	0.040	0.61	−0.023	0.034	0.50
Blood glucose	3940	0.003	0.031	0.91	−0.069	0.033	0.033	0.060	0.037	0.11	0.009	0.033	0.78
HOMA-IR	3858	0.087	0.035	0.013	−0.017	0.036	0.64	0.060	0.044	0.17	0.039	0.036	0.28
Insulin	3870	0.112	0.037	0.002	0.001	0.038	0.97	0.064	0.046	0.16	0.054	0.038	0.16
Systolic BP	3950	0.045	0.030	0.14	−0.004	0.032	0.89	0.060	0.037	0.11	0.040	0.032	0.22
Diastolic BP	3950	0.093	0.035	0.008	0.023	0.037	0.52	0.093	0.043	0.03	0.059	0.037	0.11
Total cholesterol	3940	−0.054	0.036	0.13	−0.055	0.037	0.14	−0.045	0.044	0.31	−0.042	0.038	0.27
HDL-cholesterol	3940	−0.092	0.034	0.006	0.016	0.035	0.65	−0.007	0.042	0.86	−0.054	0.036	0.13
LDL-cholesterol	3879	−0.041	0.036	0.25	−0.069	0.038	0.066	−0.084	0.044	0.060	−0.034	0.038	0.38
Triglycerides	3940	0.051	0.035	0.15	−0.007	0.037	0.85	0.073	0.044	0.093	0.036	0.037	0.34
Apolipoprotein B	3909	−0.034	0.035	0.34	−0.073	0.037	0.048	−0.056	0.043	0.20	−0.032	0.037	0.39
Apolipoprotein A-I	3903	−0.059	0.034	0.088	0.033	0.036	0.37	0.009	0.042	0.83	−0.048	0.036	0.18
		**OR**	**LL**	**UL**	**OR**	**LL**	**UL**	**OR**	**LL**	**UL**	**OR**	**LL**	**UL**
Overweight/obesity	3949	1.17	1.00	1.37	0.95	0.81	1.12	1.09	0.90	1.32	1.10	0.93	1.30
Diabetes mellitus	3919	0.91	0.71	1.16	0.95	0.74	1.21	1.17	0.89	1.54	0.90	0.70	1.17
Hypertension	3930	1.11	0.94	1.30	0.99	0.83	1.17	1.13	0.93	1.38	1.17	0.99	1.39
Hypercholesterolemia	3894	0.82	0.71	0.96	0.90	0.77	1.05	0.91	0.76	1.08	0.90	0.77	1.05
Metabolic syndrome	3938	1.07	0.92	1.25	0.99	0.84	1.17	1.21	1.00	1.46	1.11	0.94	1.31

Regression analyses using age and sex as covariates; haplotype H1 ([GAC]GTT[C]TCTG) used as reference (one minor allele, freq. 25.2%); ^§^ [imputed] and measured SNP alleles; underline: minor alleles; * standardized variables; ° raw *p* values; bold: results statistically significant after FDR correction.

**Table 5:** Associations between *NMU* SNPs and cardio-metabolic indices previously found to be significantly associated with H8 and with *NMU* CpG sites [18].

		rs14022008		rs28451532		rs11945489		rs3805383		rs6827359		rs12501006		rs73236170	
	N	β * ± SE	*p* °	β ± SE	*p*	β ± SE	*p*	β ± SE	*p*	β ± SE	*p*	β ± SE	*p*	β ± SE	*p*
*Glucose metabolism*															
Insulin	3871	-	-	0.09 ± 0.03	0.003	-	-	-	-	-	-	0.12 ± 0.06	0.048	-	-
HOMA-IR	3859	-	-	0.08 ± 0.03	0.006	-	-	-	-	-	-	0.18 ± 0.06	0.002	-	-
*Lipid-related metabolism*															
HDL-cholesterol	3941	−1.05 ± 0.47	0.025	-	-	1.09 ± 0.47	0.020	1.00 ± 0.47	0.032	−1.08 ± 0.47	0.021	-	-	-	-
Total cholesterol	3941	−1.91 ± 0.50	0.0001	-	-	2.14 ± 0.50	<0.0001	1.92 ± 0.50	0.0001	−2.17 ± 0.50	<0.0001	-	-	0.22 ± 0.07	0.002
LDL-cholesterol	3880	−2.08 ± 0.50	<0.0001	-	-	2.28 ± 0.50	<0.0001	2.07 ± 0.50	<0.0001	−2.32 ± 0.50	<0.0001	-	-	0.22 ± 0.07	0.002
Apolipoprotein B				−0.19 ± 0.07	0.006									0.19 ± 0.07	0.006

Multivariable regression analyses using all SNPs and age and sex as covariates stepwise with backward elimination (F-statistic *p*-value < 0.05); ° raw *p*-values; * standardized variables.

**Table 6:** Associations between *NMU* SNPs, CpG methylation levels, and their interactions with total and LDL-cholesterol and apolipoprotein B levels.

	Total Cholesterol (n = 1129)				LDL-Cholesterol (n = 1106)				Apolipoprotein B (n = 1115)			
	Model 1		Model 2		Model 1		Model 2		Model 1		Model 2	
	β ± SE	*p*	β ± SE	*p*	β ± SE	*p*	β ± SE	*p*	β ± SE	*p*	β ± SE	*p*
*SNPs*												
rs3805383	−0.43 ± 0.14	0.0020	-	-	−0.44 ± 0.14	0.002	-	-	-	-	-	-
rs73236170	0.42 ± 0.14	0.0030	-	-	0.48 ± 0.15	0.001	1.91 ± 0.72	0.008	-	-	2.44 ± 0.79	0.002
rs4865020	−0.4 ± 0.16	0.015	−0.3 ± 0.11	0.006	−0.43 ± 0.17	0.010	−0.39 ± 0.13	0.003	-	-	-	-
rs55796004	0.38 ± 0.16	0.016	0.29 ± 0.11	0.008	0.39 ± 0.16	0.014	0.36 ± 0.13	0.005	-	-	-	-
*CpG*												
CpG04 ^§^	-	-	-	-	0.11 ± 0.03	0.0002			0.18 ± 0.03	<0.0001	-	-
CpG05 ^§^	0.13 ± 0.03	0.0001	-	-			0.2 ± 0.09	0.019	-	-	0.38 ± 0.09	0.0001
CpG09 ^§^	−0.09 ± 0.03	0.0050	−0.09 ± 0.03	0.003	−0.11 ± 0.03	0.0005	−0.12 ± 0.03	0.0001	−0.09 ± 0.03	0.006	−0.1 ± 0.03	0.004
*Interactions*												
rs73236170 × CpG04			0.17 ± 0.04	<0.0001			-	-			-	-
rs73236170 × CpG05			-	-			−0.57 ± 0.28	0.041			−0.9 ± 0.31	0.004
rs3805383(inv) × CpG04			0.17 ± 0.04	<0.0001			0.2 ± 0.05	0.0001			0.34 ± 0.08	<0.0001
rs3805383(inv) × CpG05			-	-			-	-			−0.23 ± 0.07	0.0008
*R-square*	0.046		0.045		0.034		0.043		0.045		0.056	

Model 1: SNPs and CpG sites found to be associated in previous analyses; Model 2: Model 1 plus interactions between SNPs and CpG sites included in Model 1 found to be associated with the phenotype in single models, including the interaction and its constitutive terms. ^§^ Log-transformed variables; Rs3805383 was inverted to use as an interaction term due to its negative associations with all phenotypes in univariate models (Supplementary Table S6).

## Data Availability

The data that support the findings of this study are available from the corresponding author upon reasonable request. The data are stored in an institutional repository (https://repository.neuromed.it (accessed on 31 July 2025)) and their access is restricted by the ethical approvals and the legislation of the European Union.

## Supplementary Materials

The following supporting information can be downloaded at: https://www.mdpi.com/article/10.3390/biomedicines13081906/s1, Supplementary Table S1. Anthropometrical and biochemical variables of the sub-cohort sample (N = 4031) and the whole Moli-sani cohort population (N = 24,325). Supplementary Figure S1. Rs4865020 in the putative regulatory/promoter NMU region co-localized with a CTCF binding site region. Supplementary Figure S2. Rs3805383 in the NMU gene body tags the H3K27Ac and H3K4Me1 histone marks. Supplementary Table S2. Primers and probes of the allelic discrimination assay. Supplementary Table S3. Characteristics of the NMU SNPs and frequencies of measured genotypes. Supplementary Table S4. List of SNPs in linkage disequilibrium (LD = 1) with those SNPs analyzed in the haplotypes studied. Supplementary Table S5. Association between haplotypes with frequencies lower than 10% from both promoter and internal regions and cardio-metabolic indices. Supplementary Table S6a,b Association between promoter/internal SNPs and cardiometabolic indices. Supplementary Table S7. Association between genetic variants and CpG methylation levels.

## Abbreviations

BMI	Body mass index
CCTF	CCCTC-binding factor
CEU	Caucasian population, Utah residents with Northern and Western European ancestry
CI	Confidence interval
CVD	Cardiovascular disease
FDR	False discovery rate
GWAS	Genome-wide association study
HDL	High-density lipoprotein
HOMA-IR	Homeostatic model assessment of insulin resistance
HWE	Hardy–Weinberg equilibrium
LDL	Low-density lipoprotein
MAF	Minor allele frequency
NMU	Neuromedin U
OR	Odds ratio
SD	Standard deviation
SE	Standard error
SNP	Single nucleotide polymorphism
TSI	Tuscan population

## Appendix A. Moli-sani Study Investigators

The enrolment phase of the Moli-sani study was conducted at the Research Laboratories of the Catholic University in Campobasso (Italy). The follow-up of the Moli-sani cohort is being conducted at the Research Unit of Epidemiology and Prevention at the IRCCS Neuromed, Pozzilli, Italy.

Steering Committee: Licia Iacoviello *^#^ (Chairperson), Giovanni de Gaetano *, Maria Benedetta Donati *.
Scientific Secretariat: Chiara Cerletti * (Coordinator), Marialaura Bonaccio *, Americo Bonanni *, Simona Costanzo *°, Amalia De Curtis *, Augusto Di Castelnuovo *, Alessandro Gialluisi *^#^, Francesco Gianfagna °, Mariarosaria Persichillo *.
Safety and Ethical Committee: JosVermylen (Catholic University, Leuven, Belgio) (Chairperson), Renzo Pegoraro (Pontificia Accademia per la Vita, Roma, Italy), Antonio G. Spagnolo (Catholic University, Roma, Italy).
External Event Adjudicating Committee: Deodato Assanelli (Brescia, Italy), Livia Rago (Campobasso, Italy).
Baseline and Follow-up Data Management: Simona Costanzo *° (Coordinator), Sabatino Orlandi *, Teresa Panzera *.
Data Analysis: Augusto Di Castelnuovo * (Coordinator), Marialaura Bonaccio *, Francesca Bracone *, Simona Costanzo *°, Giuseppe Di Costanzo *, Simona Esposito *, Alessandro Gialluisi *^#^, Anwal Ghulam °, Francesco Gianfagna °, Martina Morelli *^†^, Maria Loreto MuñozVenegas *^†^, Antonietta Pepe *, Emilia Ruggiero *^§^.
Biobank, Molecular and Genetic Laboratory: Amalia De Curtis * (Coordinator), Concetta Civitillo *^†^, Alisia Cretella *^†^, Sara Magnacca *.
Recruitment Staff: Mariarosaria Persichillo * (Coordinator), Francesca Bracone *, Giuseppe Di Costanzo *, Martina Morelli *^†^.
Communication and Press Office: Americo Bonanni *.
Regional Institutions: Direzione Generale per la Salute—Regione Molise; Azienda Sanitaria Regionale del Molise (ASReM, Italy); Agenzia Regionale per la Protezione Ambientale del Molise (ARPA Molise, Italy); Molise Dati Spa (Campobasso, Italy); Offices of vital statistics of the Molise region.
Hospitals: Presidi Ospedalieri ASReM: Ospedale A. Cardarelli—Campobasso, Ospedale F. Veneziale—Isernia, Ospedale San Timoteo—Termoli (CB), Ospedale Ss. Rosario—Venafro (IS), Ospedale Vietri—Larino (CB), Ospedale San Francesco Caracciolo—Agnone (IS); Casa di Cura Villa Maria—Campobasso; Responsible Research Hospital—Campobasso; IRCCS Neuromed—Pozzilli (IS).
* Research Unit of Epidemiology and Prevention, IRCCS Neuromed, Pozzilli, Italy.
^#^ Department of Medicine and Surgery, LUM University “Giuseppe Degennaro”, Casamassima, Italy.
° Department of Medicine and Surgery, University of Insubria, Varese, Italy.
^§^ Fellow of the Fondazione Umberto Veronesi, Italy.
^†^ Fondazione Veronesi—Piattaforma UMBERTO.
Moli-sani study past investigators are available at https://www.moli-sani.org/?page_id=173 (accessed on 12 April 2025).
