# Supplementary material for: Haplotypes, Genotypes, and DNA Methylation Levels of Neuromedin U Gene Are Associated with Cardio-Metabolic Parameters: Results from the Moli-sani Study
Source: Biomedicines. 2025 Aug 5;13(8):1906. doi: 10.3390/biomedicines13081906 (PMC12383673; doi:10.3390/biomedicines13081906)
Supplement: Supplementary file 1 [file biomedicines-13-01906-s001.zip › biomedicines-3728513-supplementary.pdf]

Supplementary Material

Haplotypes, Genotypes, and DNA Methylation Levels of Neuromedin U Gene Are Associated with Cardio-Metabolic Parameters: Results from the Moli-sani Study

Noro F and Marotta A, et al.

METHODS SECTION

Supplementary Table S1. Anthropometrical and biochemical variables of the sub-cohort sample (N=4,031) and the whole Moli-sani cohort (N=24,325).

	Sub-cohort			Whole Moli-sani cohort		
**Variables**	**N**	**Mean**	**SD**	**N**	**Mean**	**SD**
Age (years)	3953	55.7	12.1	24325	55.8	12.0
Leisure-time physical activity (MET-h/day)	3953	3.2	3.8	24325	3.5	4.0
Mediterranean diet score	3938	4.3	1.6	24221	4.3	1.6
Alcohol intake (g/day)	3938	17.3	23.9	24225	16.0	22.6
Food intake (kcal/day)	3938	2109.4	683.1	24225	2079.0	667.7
BMI (kg/m^2)	3950	28.11	4.63	24308	28.06	4.78
Waist-to-hip ratio	3949	0.93	0.08	24297	0.92	0.08
Glucose (mg/dl)	3941	102.26	25.10	24174	101.48	25.4
Insulin (pmol/l)	3871	57.89	41.76	23504	60.01	44.11
HOMA-IR	3859	2.17	1.88	23370	2.24	2.4
Systolic blood pressure (mmHg)	3951	141.60	20.50	24316	140.93	20.75
Diastolic blood pressure (mmHg)	3951	82.24	9.33	24316	82.24	9.64
Total cholesterol (mg/dl)	3941	216.32	41.24	24174	213.2	41.8
LDL-cholesterol (mg/dl)	3880	132.21	35.55	23818	130.1	35.1
Apolipoprotein B (g/l)	3910	0.97	0.23	23683	0.98	0.24
HDL-cholesterol (mg/dl)	3941	58.22	14.56	24172	57.5	14.9
Apolipoprotein AI (g/l)	3904	1.54	0.31	23674	1.55	0.32
Triglycerides (mg/dl)	3941	131.97	84.85	24173	130.1	85.7
	**N**	**n**	**%**	**N**	**n**	**%**
Men	3953	1934	48.90	24325	11702	48.10
Ever smoked	3949	1983	50.20	24296	12246	50.40
Overweight or obesity	3950	2893	73.20	24308	17668	72.68
Diabetes mellitus	3920	384	9.80	24096	2349	9.75
Hypertension	3931	2280	58.00	24139	13776	57.07
Hypercholesterolemia	3895	1304	33.50	23952	7573	31.62
Metabolic syndrome	3939	1102	28.00	24167	6557	27.13
Previous CVD	3895	207	5.30	23916	1320	5.52

Supplementary Figure S1. Rs4865020 in the putative regulatory/promoter *NMU* region co-localized with a CTCF binding site region.
Among the SNPs in the *NMU* putative regulatory region, rs4865020 (4956bp upstream, depicted as a black line) co-localizes with a CTCF binding region (light-blue box) according to the ENCODE Candidate Cis-regulatory Elements (cCREs) database, and where moderate deposition of H3K4Me1 is also present. H3K4Me1, H3K4Me3, and H3K27Ac profiles (often found near regulatory elements) are displayed as colored overlaid histograms, using a scale with 150 as the maximum value for each histone track mode, which takes the highest signal in the selected region as 100% of the intensity and displays all other signals accordingly. Adapted from UCSC Browser.

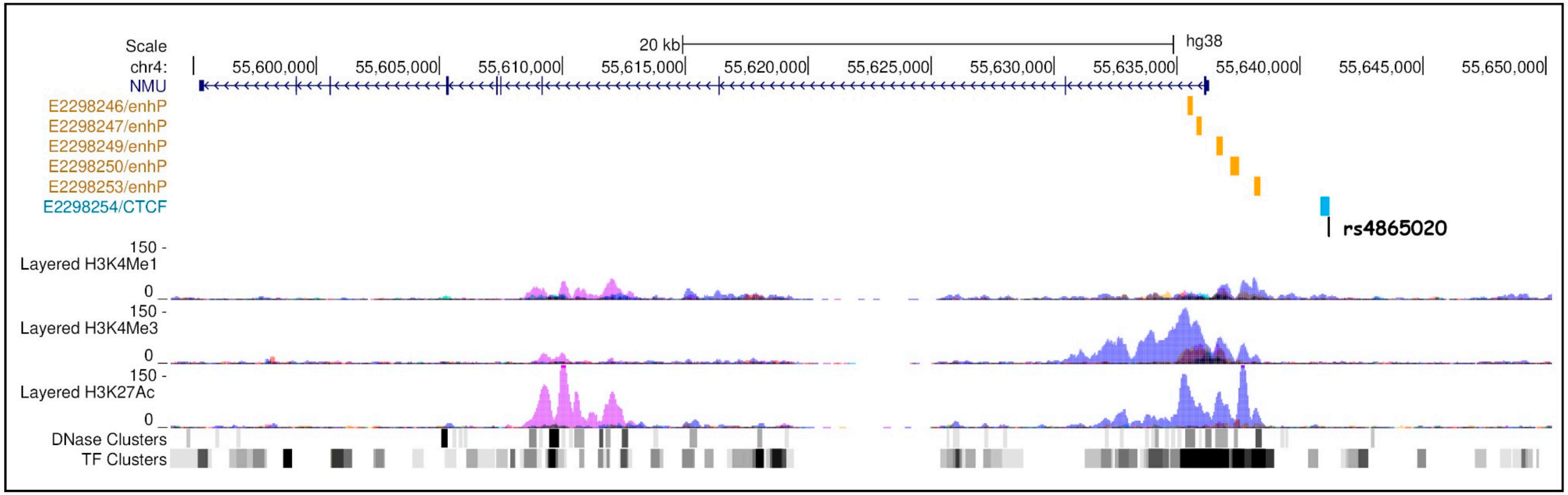

Supplementary Figure S2. Rs3805383 in the *NMU* gene body tags the H3K27Ac and H3K4Me1 histone marks.
Among the SNPs selected within the gene body, rs3805383 (synonymous variant in exon 4) tags a region where there is a high presence of the H3K27Ac histone mark and a moderate presence of the H3K4Me1 mark. This SNP is located at around 400bp from another distal enhancer element (yellow box), and it also tags a highly conserved region. The SNP is depicted as a black line. Exons are depicted as blue boxes, introns as lines. H3K4Me1, H3K4Me3, and H3K27Ac profiles (often found near regulatory elements) are displayed as colored overlaid histograms using a scale with 150 as the maximum value for each histone track. Adapted from UCSC Browser.

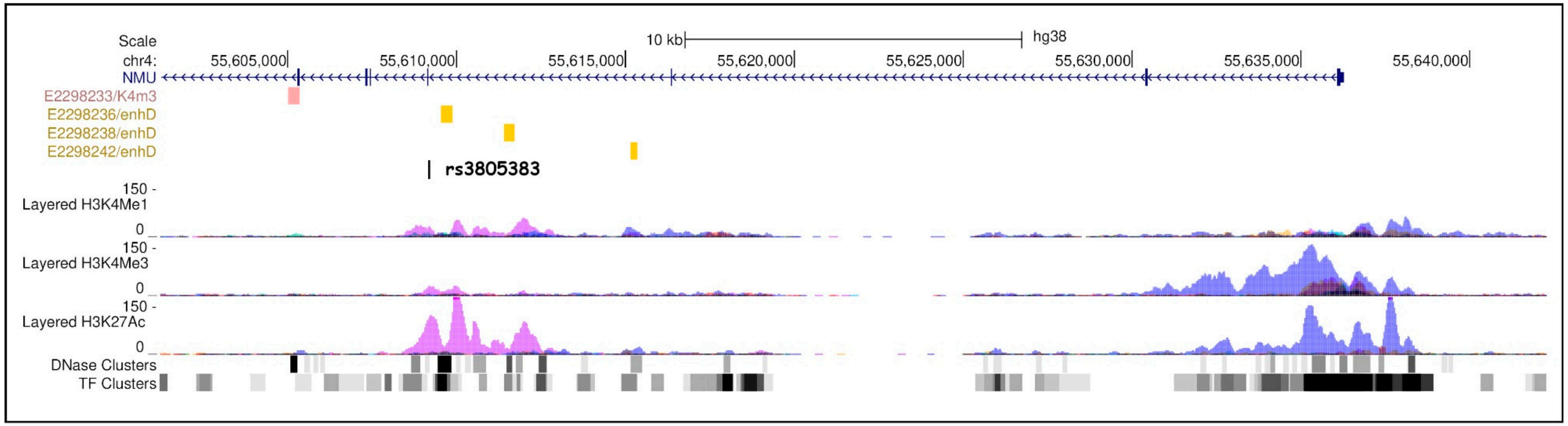

Supplementary Table S2. Primers and probes of the allelic discrimination assay.

SNP	Major/minor allele (TSI)	Primers of the assay	Probes of the assay
rs3805383	G:A*	(F) GCATTCCCATAATCATAAAGC<br>(R) AGCATAACTTACATATCTTCTCA	(FAM) TCCAGTGC**G**TTGGATG (major allele)<br>(VIC) CCTCCAGTGCATTGGAT (minor allele)
rs6827359	T*:C	(F) AACTCTCAAACGAGTTCTAA<br>(R) TTCTCTTCTAGTCTATAACTGC	(FAM) TTGCATTAACAA**A**CTTCTTGC (major allele)<br>(VIC) TAACAA**G**CTTCTTGCTCTTG (minor allele)
rs12500837	T*:C	(F) TCGATAGTGACACAAATAAAGT<br>(R) TCTTATTTTCCTGTCTTGCT	(FAM) TGTTATCCTA**A**ATAAAATGTGAACTT (major allele)<br>(VIC) TGTTATCCTA**G**ATAAAATGTGAACT (minor allele)
rs73236170	T*:C	(F) CTGTGAAATATCTCAGTGGA<br>(R) TTATAATGAATAAGAAAAAAGCTA	(FAM) ATGGGGG**A**AAATGGGC (major allele)<br>(VIC) CAATGGGGG**G**AAATGGG (minor allele)
rs62308715	C*:A	(F)ATTGCAAAGAAGTGAAATCC<br>(R) GTACAGATTGGAATGTCTGT	(FAM) ATCAAGG**G**ACCACTTCAG (major allele)<br>(VIC) TCAAGG**T**ACCACTTCAGTG (minor allele)
rs4865020	T*:C	(F) AGCCTTGTGAATAGACTAGA<br>(R) AGATTTCATTCTTTCTTCAAAGT	(FAM) CACATTAT**A**AAATTCATCCTTTTACAG (major allele)<br>(VIC) CACATTAT**G**AAATTCATCCTTTTAC (minor allele)
rs55796004	G:T*	(F) TTCAGACTGTTCTCCATAGT<br>(R) AAAGAAGAACCTTTGTAACAC	(FAM) ACTAATTT**T**CATTCCCACCAA (minor allele)<br>(VIC) ACTAATTT**G**CATTCCCACC (major allele)

*Ancestral allele
TSI: Tuscan population
(F): Forward primer; (R): reverse primer

Supplementary Table S3. Characteristics of the *NMU* SNPs and frequencies of measured genotypes.

SNP	Major/minor allele (TSI)	Major/minor allele (CEU)	Genomic position	MAF (%) TSI	MAF (%) CEU	N	Homozygotes, major		Heterozygotes		Homozygotes, minor	
							n	%	n	%	n	%
rs3805383	G:A*	G:A*	chr4:55609171	21	29	3727	2368	63.54	1181	31.69	178	4.78
rs6827359	T*:C	T*:C	chr4:55627773	46	47	3876	1015	26.19	1883	48.58	978	25.23
rs12500837	T*:C	T*:C	chr4:55627872	23	23	3730	2033	54.5	1446	38.77	251	6.73
rs73236170	T*:C	T*:C	chr4:55638944	20	20	3921	2615	66.69	1157	29.51	149	3.8
rs62308715	C*:A	C*:A	chr4:55639293	16	13	3929	2889	73.53	958	24.38	82	2.09
rs4865020	T*:C	T*:C	chr4:55641148	45	40	3922	1177	30.01	1915	48.83	830	21.16
rs57696004	G:T*	T*:G	chr4:55644087	46	49	3919	1060	27.05	1921	49.02	938	23.93

*Ancestral allele
MAF=Minor allele frequency
Genomic position (GRCh38/hg38 assembly)
CEU: Utah residents with Northern and Western European ancestry
TSI: Tuscan population

Supplementary Table S4. List of SNPs in linkage disequilibrium (LD=1) with those SNPs analyzed in the haplotypes studied.

Variant	Type of variant	SNP in linkage	Position (assembly 37)	Position (assembly 38)	MAF	Alleles	Type of variant
rs140220080	intron variant	rs73236156	chr4: 56462363	chr4: 55596196	0.068	C:G	intron variant
		rs73236157	chr4: 56465271	chr4: 55596196	0.068	A:G	
		rs55662516	chr4: 56466257	chr4: 55600090	0.068	G:A	
		rs11727729	chr4: 56466876	chr4: 55600709	0.068	T:C	
		rs11722645	chr4: 56466914	chr4: 55600747	0.068	C:T	
		rs73236159	chr4: 56475099	chr4: 55608932	0.068	G:C	
		rs73236160	chr4: 56475431	chr4: 55609264	0.068	A:G	
		rs11727831	chr4: 56476133	chr4: 55609966	0.068	C:G	
		rs73236161	chr4: 56477967	chr4: 55611800	0.068	T:C	
		rs73236162	chr4: 56478387	chr4: 55612220	0.068	C:T	
		rs73236163	chr4: 56480037	chr4: 55613870	0.068	C:G	
		rs73236164	chr4: 56481533	chr4: 55615366	0.068	C:G	
		rs11723876	chr4: 56485690	chr4: 55619523	0.068	G:A	
		rs2412668	chr4: 56493453	chr4: 55627286	0.068	C:G	
		rs73236166	chr4: 56494441	chr4: 55628274	0.068	C:T	
		rs73236167	chr4: 56495816	chr4: 55629649	0.068	C:T	
		rs11727450	chr4: 56496144	chr4: 55629977	0.068	T:C	
		rs11722402	chr4: 56496316	chr4: 55630149	0.068	C:A	
		rs73236168	chr4: 56496346	chr4: 55630179	0.068	A:T	
		rs55665531	chr4: 56496727	chr4: 55630560	0.068	C:T	

(cont.)

Supplementary Table S4 (cont.)

Variant	Type of variant	SNP in linkage	Position (assembly 37)	Position (assembly 38)	MAF	Alleles	Type of variant
rs28451532	intron variant	rs28435401	chr4: 56463823	chr4: 55597656	0.184	G:A	intron variant
		rs13434995	chr4: 56467214	chr4: 55601047	0.177	A:G	
		rs10018932	chr4: 56467866	chr4: 55601699	0.177	G:A	
		rs28756087	chr4: 56468107	chr4: 55601940	0.177	T:C	
		rs28713371	chr4: 56468422	chr4: 55602255	0.177	A:G	
		rs10011801	chr4: 56468734	chr4: 55602567	0.177	A:T	
		rs10000512	chr4: 56469022	chr4: 55602855	0.177	C:A	
		rs10011089	chr4: 56470292	chr4: 55604125	0.177	G:A	
		rs1873091	chr4: 56471433	chr4: 55605266	0.177	A:G	
		rs17725110	chr4: 56472022	chr4: 55605855	0.177	A:G	
		rs2412666	chr4: 56473359	chr4: 55607192	0.177	T:C	
		rs17725163	chr4: 56475447	chr4: 55609280	0.177	T:C	
		rs59571323	chr4: 56477354	chr4: 55611187	0.177	T:C	
		rs57178140	chr4: 56477528	chr4: 55611361	0.177	T:C	
		rs10026315	chr4: 56478362	chr4: 55612195	0.177	A:C	
		rs10017749	chr4: 56479174	chr4: 55613007	0.175	C:T	
		rs3805385	chr4: 56479988	chr4: 55613821	0.177	A:G	
		rs3805386	chr4: 56480005	chr4: 55613838	0.177	A:G	
		rs3805387	chr4: 56480008	chr4: 55613841	0.177	C:T	
		rs3805388	chr4: 56480921	chr4: 55614754	0.177	G:C	
		rs17781708	chr4: 56481069	chr4: 55614902	0.177	T:A	
		rs28473211	chr4: 56481975	chr4: 55615808	0.177	G:A	
		rs55849318	chr4: 56481993	chr4: 55615826	0.177	T:A	
		rs73236165	chr4: 56483784	chr4: 55617617	0.177	C:A	
		rs62308667	chr4: 56484402	chr4: 55618235	0.177	A:G	
		rs62308708	chr4: 56492344	chr4: 55626177	0.177	C:G	

(cont.)

Supplementary Table S4 (cont.)

		rs3805382	chr4: 56471551	chr4: 55605384	0.291	A:G	intron variant
rs11945489	intron variant	rs13147861	chr4: 56477181	chr4: 55611014	0.291	G:A	intron variant
		rs13132085	chr4: 56460085	chr4: 55593918	0.46	G:A	intergenic variant
		rs9996936	chr4: 56467639	chr4: 55601472	0.245	C:G	
		rs2412665	chr4: 56472979	chr4: 55606812	0.245	A:G	
		rs62308665	chr4: 56477797	chr4: 55611630	0.245	C:T	
rs3805383	synonymous variant	rs10026676	chr4: 56478752	chr4: 55612585	0.245	A:G	intron variant
		rs10029142	chr4: 56479134	chr4: 55612967	0.245	A:G	
		rs28564902	chr4: 56479491	chr4: 55613324	0.245	T:G	
		rs3805389	chr4: 56482750	chr4: 55616583	0.245	G:A	
		rs12498734	chr4: 56492363	chr4: 55626196	0.471	A:G	
rs6827359	intron variant	rs12499623	chr4: 56492499	chr4: 55626332	0.471	T:G	intron variant
		rs11133404	chr4: 56494321	chr4: 55628154	0.468	C:T	
		rs12509559	chr4: 56504466	chr4: 55638299	0.07	G:T	regulatory region variant
		rs73236171	chr4: 56506731	chr4: 55640564	0.07	T:A	intergenic variant
rs12501006	regulatory region variant 653 bp 5' *NMU*	rs73236172	chr4: 56513187	chr4: 55647020	0.07	C:T	intergenic variant
		rs56260406	chr4: 56515823	chr4: 55649656	0.07	C:T	intergenic variant
		rs55885309	chr4: 56516030	chr4: 55649863	0.07	G:A	intergenic variant
		rs73236173	chr4: 56516760	chr4: 55650593	0.07	G:T	intergenic variant
		rs4865021	chr4: 56508873	chr4: 55642706	0.43	C:T	
		rs12510588	chr4: 56509111	chr4: 55642944	0.43	A:T	
		rs12512220	chr4: 56509293	chr4: 55643126	0.43	T:C	
		rs62308716	chr4: 56509779	chr4: 55643612	0.43	A:T	
rs4865020	intergenic variant 4.4kb 5' of *NMU*	rs56149125	chr4: 56510321	chr4: 55644154	0.43	C:T	intergenic variant
		rs62308719	chr4: 56510573	chr4: 55644406	0.43	C:A	
		rs1027108	chr4: 56512857	chr4: 55646690	0.413	G:A	
		rs7680941	chr4: 56514087	chr4: 55647920	0.413	A:T	
		rs7697972	chr4: 56515579	chr4: 55649412	0.43	T:C	
rs55796004	intergenic variant	rs12510511	chr4: 56511059	chr4: 55644892	0.5	C:C	intergenic variant

Supplementary Table S5. Association between haplotypes with frequencies lower than 10% from both promoter and internal regions and cardio-metabolic indices. Generalized linear model using age and sex as covariates, codominant model; standardized estimates (see Supplementary Table 6 for SD values); odds ratios (ORs) and 95%CI lower limit (LL) and upper limit (UL).

		**H2 ([GAC]GTT[C]TCCT), 6.0%**			**H4 ([GAT]GCT[C]TCCT), 5.3%**			**H5 ([AAC]ATT[G]TCTT), 3.2%**			**Hrare (***********), 4.2%**		
**Dependent variable**	**Null**	**β**	**SE**	**p**	**β**	**SE**	**p**	**β**	**SE**	**p**	**β**	**SE**	**p**
BMI	3949	0.105	0.053	0.046	0.046	0.054	0.40	0.072	0.067	0.29	0.034	0.060	0.57
Waist-to-hip ratio	3948	0.006	0.048	0.90	-0.041	0.050	0.41	0.024	0.061	0.69	-0.006	0.055	0.91
Blood glucose	3940	0.030	0.045	0.51	0.037	0.048	0.43	0.039	0.062	0.53	-0.006	0.052	0.91
HOMA-IR	3858	0.056	0.050	0.27	0.014	0.054	0.79	0.272	0.069	<0.001	0.028	0.059	0.63
Insulin	3870	0.074	0.053	0.16	-0.006	0.055	0.91	0.252	0.071	<0.001	0.008	0.061	0.90
Systolic blood pressure	3950	0.104	0.045	0.021	0.031	0.047	0.51	0.037	0.057	0.51	0.076	0.052	0.14
Diastolic blood pressure	3950	0.121	0.052	0.019	0.049	0.054	0.36	0.112	0.066	0.091	0.111	0.059	0.059
Total cholesterol	3940	-0.037	0.053	0.49	-0.062	0.055	0.26	0.039	0.067	0.56	-0.035	0.060	0.57
HDL-cholesterol	3940	-0.013	0.051	0.79	-0.065	0.051	0.21	-0.070	0.063	0.27	0.001	0.057	0.99
LDL-cholesterol	3879	-0.061	0.054	0.26	-0.021	0.055	0.70	0.031	0.068	0.65	-0.041	0.061	0.50
Triglycerides	3940	0.055	0.053	0.30	-0.060	0.053	0.26	0.094	0.066	0.16	0.020	0.062	0.75
Apolipoprotein B	3909	-0.097	0.052	0.064	-0.028	0.054	0.61	0.091	0.067	0.18	-0.077	0.060	0.19
Apolipoprotein AI	3903	-0.097	0.051	0.057	-0.050	0.052	0.34	0.082	0.066	0.21	-0.074	0.058	0.20
		**OR**	**LL**	**UL**	**OR**	**LL**	**UL**	**OR**	**LL**	**UL**	**OR**	**LL**	**UL**
Overweight or obesity	3949	0.94	0.75	1.18	1.22	0.95	1.56	**1.38**	**1.01**	**1.90**	1.05	0.81	1.36
Diabetes mellitus	3919	1.01	0.71	1.43	0.82	0.57	1.20	1.26	0.82	1.92	0.94	0.62	1.42
Hypertension	3930	1.05	0.83	1.33	0.93	0.73	1.19	0.98	0.73	1.31	1.19	0.91	1.54
Hypercholesterolemia	3894	0.90	0.72	1.11	0.84	0.67	1.05	1.15	0.88	1.52	0.95	0.75	1.22
Metabolic syndrome	3938	1.13	0.90	1.43	0.96	0.75	1.22	1.19	0.89	1.59	1.04	0.80	1.36

Reference haplotype [GAC]GTT[C]TCTG, 25.2%
Green: nominally significant p-values; pink: FDR-significant p-values
Bold: nominally significant ORs

Supplementary Table S6a. Association between promoter/internal SNPs and cardiometabolic indices. Regression analysis using age and sex as covariates, codominant model; standardized estimates (see Supplementary Table 6 for SD values); odds ratios (ORs) and 95%CI lower limit (LL) and upper limit (UL).

**Dependent**		**rs140220080**			**rs28451532**			**rs11945489**			**rs3805383**		
**Continuous variables**	**N**	**β**	**SE**	**p**	**β**	**SE**	**p**	**β**	**SE**	**p**	**β**	**SE**	**p**
BMI	3950	0.012	0.057	0.83	0.043	0.029	0.13	-0.003	0.024	0.90	0.038	0.027	0.15
Waist-to-hip ratio	3949	0.032	0.052	0.55	0.045	0.026	0.090	-0.062	0.022	0.0039	0.043	0.025	0.080
Blood glucose	3941	0.006	0.050	0.90	0.012	0.025	0.64	-0.017	0.021	0.40	0.012	0.023	0.61
HOMA-IR	3871	0.043	0.058	0.47	0.087	0.029	0.0033	-0.038	0.024	0.12	0.082	0.027	0.0026
Insulin	3859	0.081	0.055	0.14	0.073	0.028	0.0089	-0.035	0.023	0.13	0.080	0.026	0.0022
Systolic blood pressure	3951	0.016	0.049	0.75	0.016	0.025	0.52	-0.010	0.020	0.62	0.018	0.023	0.44
Diastolic blood pressure	3951	0.062	0.056	0.27	0.043	0.028	0.13	-0.001	0.023	0.97	0.053	0.026	0.045
Total cholesterol	3941	0.023	0.057	0.69	-0.047	0.029	0.11	-0.019	0.024	0.42	-0.027	0.027	0.31
HDL-cholesterol	3880	0.016	0.058	0.78	-0.029	0.029	0.33	-0.034	0.024	0.16	-0.013	0.027	0.64
LDL-cholesterol	3910	0.062	0.057	0.27	-0.016	0.029	0.58	-0.030	0.023	0.21	0.004	0.027	0.89
Triglycerides	3941	-0.042	0.054	0.43	-0.081	0.027	0.0032	0.032	0.022	0.15	-0.075	0.025	0.0032
Apolipoprotein B	3904	0.067	0.056	0.23	-0.047	0.028	0.091	0.041	0.023	0.073	-0.025	0.026	0.33
Apolipoprotein AI	3941	0.048	0.056	0.40	0.031	0.029	0.28	-0.013	0.023	0.57	0.036	0.027	0.18
**Categorical variables**	**N**	**OR**	**LL**	**UL**	**OR**	**LL**	**UL**	**OR**	**LL**	**UL**	**OR**	**LL**	**UL**
Overweight or obesity	3950	1.26	0.96	1.65	1.10	0.97	1.26	0.99	0.89	1.10	**1.14**	**1.01**	**1.29**
Diabetes	3920	1.22	0.83	1.78	0.95	0.77	1.16	1.01	0.86	1.19	1.01	0.84	1.22
Hypertension	3931	0.94	0.73	1.22	1.04	0.91	1.18	0.98	0.88	1.09	1.02	0.90	1.15
Hypercholesterolemia	3895	1.15	0.91	1.46	**0.85**	**0.75**	**0.97**	0.98	0.89	1.09	0.91	0.81	1.02
Metabolic syndrome	3939	1.14	0.89	1.46	1.02	0.90	1.16	0.98	0.88	1.09	1.05	0.93	1.18

Green: nominally significant p-values; pink: FDR-significant p-values
Bold: nominally significant ORs

(cont.)

Supplementary Table S6a (cont.)

Dependent		rs6827359			rs12500837			rs12501006			rs73236170		
**Continuous variables**	**N**	**β**	**SE**	**p**	**β**	**SE**	**p**	**β**	**SE**	**p**	**β**	**SE**	**p**
BMI	3950	0.021	0.022	0.33	-0.019	0.025	0.45	0.025	0.059	0.67	0.042	0.028	0.13
Waist-to-hip ratio	3949	-0.029	0.020	0.15	-0.062	0.023	0.0073	0.050	0.055	0.36	0.039	0.026	0.13
Blood glucose	3941	-0.009	0.019	0.65	-0.022	0.022	0.32	0.075	0.052	0.15	-0.009	0.025	0.72
HOMA-IR	3871	0.017	0.023	0.46	-0.030	0.026	0.24	0.113	0.061	0.061	0.070	0.029	0.015
Insulin	3859	0.012	0.021	0.59	-0.032	0.024	0.19	0.171	0.058	**0.0029**	0.056	0.027	0.041
Systolic blood pressure	3951	0.001	0.019	0.95	-0.004	0.021	0.86	-0.002	0.051	0.96	0.026	0.024	0.28
Diastolic blood pressure	3951	0.026	0.022	0.23	0.008	0.025	0.75	0.050	0.059	0.390	0.049	0.028	0.076
Total cholesterol	3941	-0.042	0.022	0.056	-0.018	0.025	0.49	-0.001	0.060	0.99	0.000	0.028	0.99
HDL-cholesterol	3880	-0.045	0.022	0.044	-0.045	0.025	0.080	-0.016	0.060	0.80	0.017	0.029	0.56
LDL-cholesterol	3910	-0.034	0.022	0.12	-0.037	0.025	0.14	0.050	0.059	0.40	0.018	0.028	0.52
Triglycerides	3941	-0.018	0.021	0.38	0.051	0.024	0.031	-0.043	0.056	0.44	-0.058	0.027	0.030
Apolipoprotein B	3904	0.008	0.021	0.69	0.054	0.024	0.028	0.082	0.058	0.16	-0.036	0.027	0.18
Apolipoprotein AI	3941	0.007	0.022	0.76	-0.002	0.025	0.93	0.060	0.059	0.31	0.027	0.028	0.34
**Categorical variables**	**N**	**OR**	**LL**	**UL**	**OR**	**LL**	**UL**	**OR**	**LL**	**UL**	**OR**	**LL**	**UL**
Overweight or obesity	3950	1.04	0.94	1.16	0.92	0.82	1.03	1.22	0.92	1.61	1.11	0.98	1.27
Diabetes	3920	0.98	0.84	1.14	1.11	0.93	1.31	1.43	0.98	2.08	0.91	0.75	1.11
Hypertension	3931	1.00	0.91	1.11	1.02	0.91	1.15	0.91	0.69	1.18	1.08	0.95	1.23
Hypercholesterolemia	3895	**0.90**	**0.82**	**0.99**	1.00	0.90	1.11	1.16	0.90	1.49	0.91	0.81	1.03
Metabolic syndrome	3939	1.00	0.90	1.10	1.01	0.90	1.13	1.22	0.94	1.59	1.00	0.88	1.13

Green: nominally significant p-values; pink: FDR-significant p-values
Bold: nominally significant ORs

(cont.)

Supplementary Table S6a (cont.)

Dependent		rs62308715			rs4865020			rs55796004		
**Continuous variables**	**N**	**β**	**SE**	**p**	**β**	**SE**	**p**	**β**	**SE**	**p**
BMI	3950	0.008	0.032	0.80	0.023	0.022	0.30	0.026	0.022	0.23
Waist-to-hip ratio	3949	-0.005	0.029	0.86	-0.016	0.020	0.44	-0.008	0.020	0.67
Blood glucose	3941	0.007	0.028	0.81	-0.032	0.019	0.10	-0.019	0.019	0.34
HOMA-IR	3871	0.008	0.033	0.81	0.014	0.023	0.54	0.032	0.023	0.15
Insulin	3859	-0.001	0.031	0.98	0.002	0.022	0.92	0.029	0.021	0.17
Systolic blood pressure	3951	0.006	0.027	0.84	0.013	0.019	0.50	0.012	0.019	0.53
Diastolic blood pressure	3951	0.000	0.031	0.99	0.025	0.022	0.25	0.032	0.022	0.14
Total cholesterol	3941	-0.005	0.032	0.88	-0.022	0.022	0.33	-0.026	0.022	0.24
HDL-cholesterol	3880	0.009	0.032	0.78	-0.013	0.022	0.55	-0.021	0.022	0.34
LDL-cholesterol	3910	0.006	0.032	0.85	-0.028	0.022	0.21	-0.025	0.022	0.25
Triglycerides	3941	-0.030	0.030	0.31	-0.018	0.021	0.38	-0.023	0.021	0.26
Apolipoprotein B	3904	-0.036	0.031	0.24	-0.023	0.021	0.29	-0.010	0.021	0.64
Apolipoprotein AI	3941	0.013	0.031	0.67	-0.009	0.022	0.67	0.001	0.022	0.97
**Categorical variables**	**N**	**OR**	**LL**	**UL**	**OR**	**LL**	**UL**	**OR**	**LL**	**UL**
Overweight or obesity	3950	1.06	0.92	1.23	1.00	0.90	1.10	1.02	0.92	1.12
Diabetes	3920	0.90	0.72	1.12	0.92	0.79	1.07	0.98	0.85	1.15
Hypertension	3931	1.13	0.97	1.31	0.97	0.88	1.08	0.96	0.87	1.06
Hypercholesterolemia	3895	1.00	0.87	1.14	**0.91**	**0.83**	**1.00**	0.93	0.85	1.02
Metabolic syndrome	3939	1.05	0.91	1.21	0.95	0.86	1.05	0.98	0.88	1.08

Bold: nominally significant ORs

Supplementary Table S6b. Association between promoter/internal SNPs and cardiometabolic indices. Regression analysis using age, sex, and all genotypes as covariates with backward elimination (only associations with p<0.05 were retained in the model), codominant model; standardized estimates (see Supplementary Table 6 for SD values); odds ratios (ORs) and 95%CI lower limit (LL) and upper limit (UL).

**Dependent**		**rs140220080**			**rs28451532**			**rs11945489**			**rs3805383**		
**Continuous variables**	**N**	**β**	**SE**	**p**	**β**	**SE**	**p**	**β**	**SE**	**p**	**β**	**SE**	**p**
BMI	3950												
Waist-to-hip ratio	3949							-0.062	0.022	0.0039			
Blood glucose	3941	-0.183	0.092	0.046									
HOMA-IR	3871				0.089	0.029	0.0026						
Insulin	3859				0.077	0.028	0.0062						
Systolic blood pressure	3951												
Diastolic blood pressure	3951												
Total cholesterol	3941	-1.914	0.498	0.0001				2.144	0.497	2.E-05	1.916	0.495	0.0001
HDL-cholesterol	3880	-2.076	0.499	3.E-05				2.277	0.498	5.E-06	2.073	0.497	3.E-05
LDL-cholesterol	3910				-0.191	0.069	0.0059						
Triglycerides	3941	-1.054	0.469	0.025				1.088	0.468	0.020	1.001	0.467	0.032
Apolipoprotein B	3904												
Apolipoprotein AI	3941												
**Categorical variables**	**N**	**OR**	**LL**	**UL**	**OR**	**LL**	**UL**	**OR**	**LL**	**UL**	**OR**	**LL**	**UL**
Overweight or obesity	3950							1.29	1.04	1.61	1.16	1.01	1.32
Diabetes	3920												
Hypertension	3931												
Hypercholesterolemia	3895				0.85	0.75	0.97						
Metabolic syndrome	3939												

(cont.)

Supplementary Table S6b (cont.)

Dependent		rs6827359			rs12500837			rs12501006			rs73236170		
**Continuous variables**	**N**	**β**	**SE**	**p**	**β**	**SE**	**p**	**β**	**SE**	**p**	**β**	**SE**	**p**
BMI	3950												
Waist-to-hip ratio	3949												
Blood glucose	3941							0.236	0.096	0.014			
HOMA-IR	3871							0.120	0.061	0.048			
Insulin	3859							0.177	0.058	0.0021			
Systolic blood pressure	3951												
Diastolic blood pressure	3951												
Total cholesterol	3941	-2.172	0.496	1.E-05							0.218	0.069	0.0015
HDL-cholesterol	3880	-2.317	0.497	3.E-06							0.219	0.070	0.0016
LDL-cholesterol	3910										0.187	0.067	0.0056
Triglycerides	3941	-1.077	0.467	0.021									
Apolipoprotein B	3904				0.054	0.024	0.028						
Apolipoprotein AI	3941												
**Categorical variables**	**N**	**OR**	**LL**	**UL**	**OR**	**LL**	**UL**	**OR**	**LL**	**UL**	**OR**	**LL**	**UL**
Overweight or obesity	3950				0.76	0.60	0.95						
Diabetes	3920												
Hypertension	3931												
Hypercholesterolemia	3895												
Metabolic syndrome	3939												

(cont.)

Supplementary Table S6b (cont.)

Dependent		rs62308715			rs4865020			rs55796004		
**Continuous variables**	**N**	**β**	**SE**	**p**	**β**	**SE**	**p**	**β**	**SE**	**p**
BMI	3950									
Waist-to-hip ratio	3949									
Blood glucose	3941									
HOMA-IR	3871									
Insulin	3859									
Systolic blood pressure	3951									
Diastolic blood pressure	3951									
Total cholesterol	3941									
HDL-cholesterol	3880									
LDL-cholesterol	3910									
Triglycerides	3941									
Apolipoprotein B	3904									
Apolipoprotein AI	3941									
**Categorical variables**	**N**	**OR**	**LL**	**UL**	**OR**	**LL**	**UL**	**OR**	**LL**	**UL**
Overweight or obesity	3950									
Diabetes	3920				0.66	0.47	0.95	1.42	1.00	2.02
Hypertension	3931									
Hypercholesterolemia	3895									
Metabolic syndrome	3939									

Supplementary Table S7. Association between genetic variants and CpG methylation levels. Regression analysis using age and sex as covariates, codominant model; standardized estimates (see Marotta et al Epigenetics 2021 [Ref 23 in full-text] for SD values).

		**rs140220080**			**rs28451532**			**rs11945489**			**rs3805383**		
Dependent	N	**β**	**SE**	**p**	**β**	**SE**	**p**	**β**	**SE**	**p**	**β**	**SE**	**p**
NMU76 CpG03	1180	0.051	0.088	0.56	0.019	0.053	0.73	-0.040	0.043	0.35	0.031	0.049	0.52
NMU76 CpG04	1197	0.105	0.087	0.23	-0.012	0.053	0.82	-0.039	0.043	0.37	0.022	0.048	0.65
NMU76 CpG05	1164	0.018	0.082	0.82	0.054	0.050	0.28	-0.032	0.040	0.43	0.050	0.045	0.27
NMU76 CpG09	1205	-0.019	0.087	0.83	0.009	0.053	0.86	0.050	0.042	0.24	0.002	0.048	0.97

		**rs6827359**			**rs12500837**			**rs12501006**			**rs73236170**		
Dependent	N	**β**	**SE**	**p**	**β**	**SE**	**p**	**β**	**SE**	**p**	**β**	**SE**	**p**
NMU76 CpG03	1180	-0.025	0.040	0.54	-0.035	0.046	0.45	0.053	0.094	0.58	0.002	0.052	0.97
NMU76 CpG04	1197	-0.041	0.040	0.31	-0.044	0.046	0.34	0.140	0.093	0.13	-0.042	0.052	0.42
NMU76 CpG05	1164	0.003	0.038	0.94	-0.018	0.043	0.67	0.011	0.088	0.90	0.042	0.049	0.39
NMU76 CpG09	1205	0.049	0.040	0.22	0.039	0.045	0.39	-0.016	0.093	0.87	-0.009	0.051	0.86

		**rs62308715**			**rs4865020**			**rs55796004**			**H8**		
Dependent	N	**β**	**SE**	**p**	**β**	**SE**	**p**	**β**	**SE**	**p**	**β**	**SE**	**p**
NMU76 CpG03	1180	0.029	0.057	0.61	-0.017	0.040	0.66	-0.009	0.039	0.83	0.018	0.054	0.73
NMU76 CpG04	1197	-0.015	0.056	0.80	-0.024	0.040	0.54	0.002	0.039	0.96	-0.025	0.054	0.64
NMU76 CpG05	1164	-0.005	0.053	0.93	0.002	0.037	0.95	0.004	0.037	0.92	0.053	0.050	0.29
NMU76 CpG09	1205	0.070	0.056	0.21	0.009	0.039	0.81	0.010	0.039	0.81	0.008	0.053	0.88

(cont.)

Supplementary Table S7 (cont.)

Dependent	N	H7			H6			H5			H4		
		**β**	**SE**	**p**	**β**	**SE**	**p**	**β**	**SE**	**p**	**β**	**SE**	**p**
NMU76 CpG03	1180	-0.017	0.065	0.79	-0.027	0.056	0.63	0.080	0.097	0.41	-0.063	0.086	0.47
NMU76 CpG04	1197	-0.024	0.065	0.71	-0.041	0.055	0.46	0.134	0.097	0.16	-0.002	0.086	0.99
NMU76 CpG05	1164	-0.003	0.061	0.96	-0.014	0.052	0.78	0.033	0.091	0.72	-0.087	0.081	0.28
NMU76 CpG09	1205	0.004	0.065	0.95	0.063	0.055	0.25	-0.024	0.096	0.80	0.043	0.085	0.61

Dependent	N	H3			H2			H1			Rare Haplotypes		
		**β**	**SE**	**p**	**β**	**SE**	**p**	**β**	**SE**	**p**	**β**	**SE**	**p**
NMU76 CpG03	1180	0.032	0.057	0.57	0.032	0.090	0.72	-0.014	0.047	0.77	-0.013	0.107	0.91
NMU76 CpG04	1197	-0.012	0.056	0.83	0.082	0.089	0.35	0.011	0.047	0.82	0.023	0.107	0.83
NMU76 CpG05	1164	-0.003	0.053	0.96	0.017	0.083	0.84	-0.012	0.044	0.79	-0.005	0.099	0.96
NMU76 CpG09	1205	0.068	0.056	0.22	-0.123	0.088	0.16	-0.068	0.046	0.14	-0.023	0.106	0.83
